# Impact of metabolic phenotype and alcohol consumption on mortality risk in metabolic dysfunction-associated fatty liver disease: a population-based cohort study

**DOI:** 10.1038/s41598-024-63453-6

**Published:** 2024-06-03

**Authors:** Phunchai Charatcharoenwitthaya, Khemajira Karaketklang, Wichai Aekplakorn

**Affiliations:** 1 Division of Gastroenterology, Department of Medicine, Faculty of Medicine Siriraj Hospital, Mahidol University, Bangkok, 10700 Thailand; 2 Department of Community Medicine, Faculty of Medicine Ramathibodi Hospital, Mahidol University, Bangkok, Thailand

**Keywords:** Alcohol consumption, Type 2 diabetes, Metabolic phenotype, MAFLD, Mortality, Endocrinology, Gastroenterology

## Abstract

Patients with metabolic dysfunction-associated fatty liver disease (MAFLD) often present with concomitant metabolic dysregulation and alcohol consumption, potentially leading to distinct clinical outcomes. We analyzed data from 8043 participants with MAFLD in the Thai National Health Examination Survey with linked mortality records. According to the MAFLD criteria, 1432 individuals (17.2%) were categorized as having the diabetes phenotype, 5894 (71.0%) as the overweight/obesity phenotype, and 978 (11.8%) as the lean metabolic phenotype. Over 71,145 person-years, 916 participants died. Using Cox proportional hazard models adjusting for physiological, lifestyle, and comorbid factors, both diabetes (adjusted hazards ratio [aHR] 1.59, 95% CI 1.18–2.13) and lean metabolic phenotypes (aHR 1.28, 95% CI 1.01–1.64) exhibited significantly higher mortality risk compared to the overweight/obesity phenotype. A J-shaped relationship was observed between daily alcohol consumption and the risk of all-cause mortality. Daily alcohol intake exceeding 50 g for women and 60 g for men increased the all-cause mortality risk among MAFLD individuals with the lean metabolic phenotype (aHR 3.39, 95% CI 1.02–11.29). Our study found that metabolic phenotype and alcohol consumption have interactive effects on the risk of all-cause mortality in patients with MAFLD, indicating that evaluating both factors is crucial for determining prognostic outcomes and management strategies.

## Introduction

Metabolic dysfunction-associated fatty liver disease (MAFLD) is a fatty liver disease associated with metabolic risk factors^1^. Several studies demonstrate that individuals with MAFLD face an increased risk of cardiovascular disease and mortality^1–5^. Notably, MAFLD comprises three distinct subtypes: overweight and obese individuals, those with type 2 diabetes, and lean patients with two metabolic risk factors^1^. Due to this heterogeneity, further studies are required to delineate patient outcomes within each MAFLD subtype.

The definition of MAFLD is exclusively based on a set of positive criteria, regardless of the presence of other concurrent liver diseases. Thus, individuals who consume alcohol can have MAFLD (dual etiology liver disease) if they meet the MAFLD criteria, allowing for a more holistic assessment of the patient. Approximately 25% of MAFLD patients are at risk of alcohol-related liver damage^6^. This subgroup differs from those with predominant alcohol-associated liver disease without evidence of metabolic dysregulation^7^. The safe limit of alcohol intake remains a subject of debate, with some studies suggesting that even low alcohol consumption in individuals with fatty liver is associated with an increased risk of disease progression, further contributing to advanced liver disease and cancer^8^. Moreover, there is evidence indicating a synergistic, detrimental effect on liver disease progression when alcohol use is combined with features of metabolic syndrome^9,10^. Therefore, understanding the prognosis of individuals with coexisting metabolic dysfunction and alcohol consumption is crucial for implementing appropriate management strategies. In our study of a large nationwide community-based cohort, we examined the association between metabolic phenotypes and mortality risk in MAFLD patients with varying degrees of alcohol consumption.

## Methods

### Study population

This study is a longitudinal cohort comprising participants recruited from the Fourth National Health Examination Survey (NHES-IV) conducted in Thailand from 2008 to 2009. The NHES-IV is a national survey program designed to assess the general health and nutrition status of non-institutionalized Thai civilians, utilizing a complex, stratified, clustered multistage probability sampling design^11^. Of 18,323 persons aged 18 years and older, we excluded subjects with body mass index (BMI) less than 18.5 kg/m^2^ (n = 1689) and those with missing data (n = 506) on alcohol intake, BMI, and laboratory testing, including fasting glucose, high-density lipoprotein cholesterol (HDL-C), and triglycerides. Of the remaining population (n = 16,128), 8635 individuals with hepatic steatosis were identified by the Lipid Accumulation Product (LAP)^12^. According to the diagnostic criteria proposed by an international expert panel^1^, 8304 participants with metabolic risk factors and hepatic steatosis were classified as MAFLD. The study was conducted following the Declaration of Helsinki and received approval from the Siriraj Institutional Review Board. All participants provided written informed consent to participate in NHES-IV.

### Definition of fatty liver disease

We utilized a validated LAP score as a diagnostic tool for identifying hepatic steatosis^12^. LAP was calculated using the formulae for women (waist circumference (WC)_[cm] _− 58) ×  (triglyceride_[mmol/L]_) and men (WC_[cm] _− 65) ×  (triglyceride_[mmol/L]_)^12^. The formulas include minimum WC values that are specific to each sex. Subjects were considered to have hepatic steatosis if their LAP score was ≥ 30.5 in men and ≥ 23.0 in women. The accuracy of the LAP score in diagnosing fatty liver disease was evaluated in a general population-based cohort of 40,459 Chinese subjects aged ≥ 18 years who participated in annual physical examinations using ultrasound as the reference standard^12^. The LAP score demonstrated predictive capabilities, with an area under the curve value of 0.843 (95% confidence interval [CI] 0.837–0.849) for men and 0.887 (95% CI 0.882–0.892) for women in identifying hepatic steatosis^12^.

MAFLD was diagnosed as the presence of hepatic steatosis with at least one of the following criteria: (1) overweight or obese (body mass index ≥ 23 kg/m^2^ according to the Asian-specific criteria); (2) diabetes mellitus (fasting glucose of ≥ 126 mg/dL and/or treatment with antidiabetic agents); and (3) at least two cardiometabolic risk abnormalities, consisting of central obesity (WC ≥ 80 cm in women and ≥ 90 cm in men for Asians), hypertension (blood pressure ≥ 130/85 mmHg or treatment with antihypertensive drugs), hyperglycemia (fasting glucose ≥ 100 mg/dL), hypertriglyceridemia (fasting triglycerides ≥ 150 mg/dL), and low HDL-C (< 40 mg/dL in men, < 50 mg/dL in women)^1^.

Based on the multi-society Delphi consensus statement on new fatty liver disease nomenclature, individuals with hepatic steatosis are classified into three main groups according to their cardiometabolic status and alcohol consumption^13^. Metabolic dysfunction-associated steatotic liver disease (MASLD) was defined as hepatic steatosis accompanied by at least one cardiometabolic factor and daily alcohol intake < 20 g for women and < 30 g for men. Metabolic dysfunction- and alcohol-associated liver disease (MetALD) was characterized by hepatic steatosis with at least one cardiometabolic factor and daily alcohol intake ranging from 20 to 50 g for women and 30–60 g for men. Alcohol-associated liver disease (ALD) was identified as hepatic steatosis and daily alcohol intake > 50 g for women and > 60 g for men.

### Data collection measurements

To collect data, research nurses performed face-to-face interviews using standardized questions. Alcohol consumption was determined using self-reported questionnaire items concerning the frequency and amount of alcohol consumed per day in the 12 months prior to the examination. Alcohol use was classified into four categories: abstinence or light drinking (< 10 g/day), moderate drinking (10 to < 20 g/day for women and 10 to < 30 g/day for men), risk drinking (20–50 g/day for women and 30–60 g/day for men), and heavy drinking (> 50 g/day for women and > 60 g/day for men). Smoking status was categorized as never, former, or current. The Global Physical Activity Questionnaire was used to assess physical activity. High leisure-time physical activity (LTPA) refers to moderate or vigorous-intensity activities that last for at least 20 min at a time, at least three times per week. The Charlson Comorbidity Index (CCI) score was calculated for each patient to assess comorbidity^14^.

Weight, height, and WC were measured according to standard protocols. BMI was computed by dividing weight (kg) by the square of standing height (m^2^). Individuals were classified as lean (BMI 18.5–22.9 kg/m^2^), overweight (BMI 23–24.9 kg/m^2^), and obese (BMI ≥ 25 kg/m^2^) following the Asian-specific criteria^15^. Blood pressure was taken with a standard automatic blood pressure monitor. Handgrip strength was assessed with a digital dynamometer as a proxy for muscle strength. After an overnight fast, blood samples were drawn from an antecubital vein, and various parameters, such as fasting plasma glucose, total cholesterol, low-density lipoprotein cholesterol (LDL-C), HDL-C, and triglyceride levels, were analyzed. The NHES data was linked to the Ministry of Interior's National Civil Registration and Vital Statistics System to determine the mortality status of Thai residents.

### Statistical analyses

All analyses were weighted to account for the complex sampling design using STATA version 14.0 (StataCorp LP, College Station, Texas, USA). The χ^2^ test and one-way analysis of variance were employed to compare the baseline characteristics of participants. A post hoc multiple comparison analysis was performed with Bonferroni correction. The Kaplan–Meier method was used to estimate the overall survival of subjects with MAFLD, stratified by disease subtypes (diabetes, overweight/obesity, and lean metabolic disorder). The log-rank test was performed to compare survival distributions between the groups.

Overall mortality rates were determined and reported as deaths per 1000 person-years at risk. Cox proportional hazard models were used to estimate adjusted hazard ratios (aHRs) for overall mortality. These models were adjusted for age and sex, with further adjustments for education, LTPA, CCI, smoking status, alcohol intake (for models related to metabolic phenotypes), and handgrip strength. The penalized spline smoothing method was used with multivariable adjustment to investigate the association between average daily alcohol consumption and all-cause mortality. P-values for interaction were calculated to assess the relationship between metabolic phenotype and alcohol consumption on all-cause mortality. The combined effect of average alcohol consumption and metabolic phenotype on all-cause mortality was investigated, grouping individuals based on categorized average daily alcohol consumption (abstinence to light, moderate, risk, and heavy drinking) and metabolic phenotypes (overweight/obesity, type 2 diabetes, and lean metabolic disorder). Additionally, we conducted an analysis to assess the risk of all-cause mortality in individuals with MASLD, MetALD, and ALD.

## Results

### Study population

**Figure 1:**
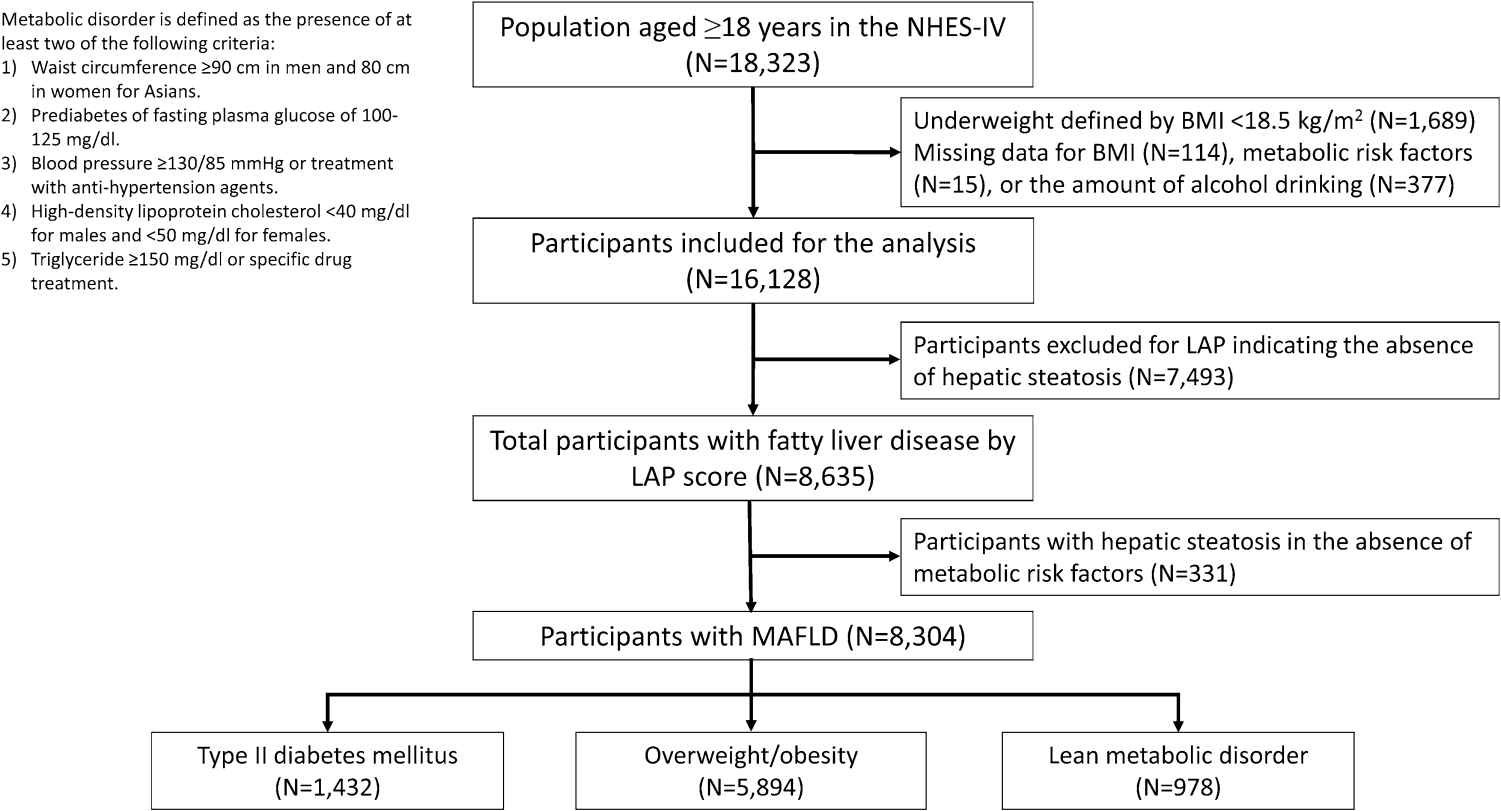
Flow chart of the study population.

According to the MAFLD definition, 3091 men and 5213 women were diagnosed with MAFLD (Fig. 1). The mean age at baseline was 47.9 ± 12.9 years, and the mean BMI was 27.0 ± 4.0 kg/m^2^. A total of 1432 subjects (17.2%) with MAFLD who had fasting glucose levels ≥ 126 mg/dl or a history of type 2 diabetes mellitus were classified as having the diabetes phenotype. Another 5894 subjects (71.0%) without diabetes but with a BMI ≥ 23 kg/m^2^ were categorized as having the overweight/obesity phenotype. Lastly, 978 lean individuals (11.8%) with two metabolic risk factors among the non-diabetic population were classified as having the lean metabolic phenotype.

### Characteristics of participants with MAFLD according to metabolic phenotypes

The baseline characteristics of the study population are presented in Table 1. Among the three MAFLD phenotypes, participants in the diabetes group were the oldest, had the highest CCI score, the highest prevalence of hyperglycemia and hypertension, and the highest levels of plasma glucose and serum creatinine. The diabetes MAFLD group included fewer participants with high levels of LTPA, hypertriglyceridemia, and low HDL-C. In contrast, those in the overweight/obesity group were significantly younger, more likely to have higher education, had the highest proportion of high LTPA, the highest mean value of handgrip strength, the lowest CCI score, and the highest mean levels of total cholesterol, LDL-C, HDL-C, and hemoglobin. The lean metabolic group had the lowest mean BMI, the lowest prevalence of hypertension and central obesity, the highest prevalence of hypertriglyceridemia and low HDL-C, and the lowest mean levels of plasma glucose and LDL-C. The diabetes and lean metabolic phenotypes were older and had weaker handgrip strength than the overweight/obesity group.

**Table 1:** Baseline characteristics of the patients with different MAFLD subtypes.

Characteristics	MAFLD				*P* value	Multiple comparison
	Overall	Overweight/obesity^(a)^	Lean metabolic disorder^(b)^	Type II diabetes^(c)^		
Number (%)	8304	5894 (71.0)	978 (11.8)	1432 (17.2)		
Age (years)	47.9 ± 12.9	46.4 ± 12.3	50.9 ± 15.4	55.1 ± 11.8	< 0.001	a $\ne$ b, a $\ne$ c
Male gender, n (%)	3091 (38.9)	2243 (40.3)	325 (34.5)	523 (33.9)	0.077	
Education, n (%)					< 0.001	a $\ne$ b, a $\ne$ c
Primary	547 (3.9)	316 (3.1)	109 (8.3)	122 (5.0)		
Secondary	5601 (67.8)	3867 (66.0)	702 (72.5)	1032 (74.7)		
Higher	2136 (28.3)	1698 (30.9)	163 (19.2)	275 (20.3)		
Body mass index (kg/m^2^)	27.0 ± 4.0	27.7 ± 3.6	21.7 ± 1.1	27.4 ± 4.9	< 0.001	a $\ne$ b $\ne$ c
Alcohol use (g/day)	21.3 ± 58.8	23.1 ± 61.5	17.4 ± 36.8	13.8 ± 43.3	< 0.001	a ≠ b, a ≠ c
Smoking status, n (%)					0.104	
Never	5850 (70.5)	4206 (71.0)	639 (65.0)	1005 (72.2)		
Former	1181 (11.4)	789 (11.4)	146 (11.4)	246 (11.7)		
Current	1273 (18.1)	899 (17.6)	193 (23.6)	181 (16.1)		
High LTPA, n (%)	6190 (81.7)	4505 (82.8)	679 (78.8)	1006 (77.2)	0.018	a $\ne$ c
Charlson Comorbidity index	0.98 ± 0.63	0.83 ± 0.53	0.89 ± 0.68	2.01 ± 0.80	< 0.001	a ≠ b ≠ c
Metabolic risk factor, n (%)						
Hyperglycemia	2830 (26.7)	1196 (16.6)	202 (16.8)	1432 (100)	< 0.001	a $\ne$ c, b $\ne \text{c}$
Hypertension	5041 (49.5)	3410 (48.2)	569 (41.7)	1062 (64.9)	< 0.001	a $\ne$ b $\ne$ c
Hypertriglyceridemia	5254 (65.3)	3476 (61.6)	825 (87.7)	953 (68.7)	< 0.001	a $\ne$ b $\ne$ c
Low HDL-C	5313 (65.0)	3566 (62.0)	763 (82.1)	984 (68.8)	< 0.001	a $\ne$ b $\ne$ c
Central obesity	6852 (82.1)	5156 (87.2)	438 (39.7)	1258 (88.3)	< 0.001	a $\ne$ b, a $\ne$ c
Glucose (mg/dL)	95.7 ± 33.9	87.4 ± 12.8	87.1 ± 13.5	156.2 ± 64.4	< 0.001	a $\ne$ c, b $\ne$ c
Total cholesterol (mg/dL)	218.8 ± 44.7	220.5 ± 43.5	209.9 ± 49.8	216.0 ± 46.0	< 0.001	a $\ne$ b $\ne$ c
LDL-C (mg/dL)	134.5 ± 41.3	137.1 ± 40.1	121.6 ± 46.7	129.3 ± 41.3	< 0.001	a $\ne$ b, a $\ne$ c
HDL-C (mg/dL)	43.3 ± 10.0	43.8 ± 9.9	40.6 ± 9.4	42.6 ± 10.4	< 0.001	a $\ne$ b $\ne$ c
Triglycerides (mg/dL)	211.0 ± 128.2	202.4 ± 117.9	252.1 ± 159.8	228.8 ± 148.2	< 0.001	a $\ne$ b $\ne$ c
Hemoglobin (g/dL)	13.4 ± 1.8	13.5 ± 1.8	13.0 ± 1.7	12.9 ± 1.8	< 0.001	a $\ne$ b, a $\ne$ c
Platelet (× 10^9^/cumm)	287 ± 73	287 ± 72	290 ± 74	284 ± 78	0.467	
Creatinine (mg/dL)	0.88 ± 0.29	0.86 ± 0.23	0.86 ± 0.32	1.00 ± 0.49	< 0.001	a $\ne$ b $\ne$ c
Handgrip strength (kg)	30.7 ± 9.7	31.7 ± 9.9	27.9 ± 8.3	27.6 ± 8.8	< 0.001	a $\ne$ b, a $\ne$ c

BMI, body mass index; BP, blood pressure; LDL-C, low-density lipoprotein cholesterol; LTPA; leisure time physical activity; HDL-C, high-density lipoprotein cholesterol; MAFLD, metabolic-associated fatty liver disease.

Data are presented as the mean ± standard deviation or number (percentage).

The χ^2^ test and one-way analysis of variance were used to compare the characteristics of the study participants. Post hoc multiple comparison analysis was performed with Bonferroni correction.

a ≠ b ≠ c: There is statistical difference among type II diabetes (a), overweight/obesity (b), and lean metabolic disorder (c).

a ≠ b, a ≠ c: There is statistical difference between type II diabetes (a) and overweight/obesity (b) and between Type II diabetes (a) and lean metabolic disorder (c), while there is no statistical difference between overweight/obesity (b) and lean metabolic disorder (c).

b ≠ c: There is statistical difference between overweight/obesity (b) and lean metabolic disorder (c).

a ≠ b, b ≠ c: There is statistical difference between type II diabetes (a) and overweight/obesity (b) and between overweight/obesity (b) and lean metabolic disorder (c), while there is no statistical difference between Type II diabetes (a) and lean metabolic disorder (c).

### All-cause mortality of MAFLD participants according to metabolic phenotypes

**Figure 2:**
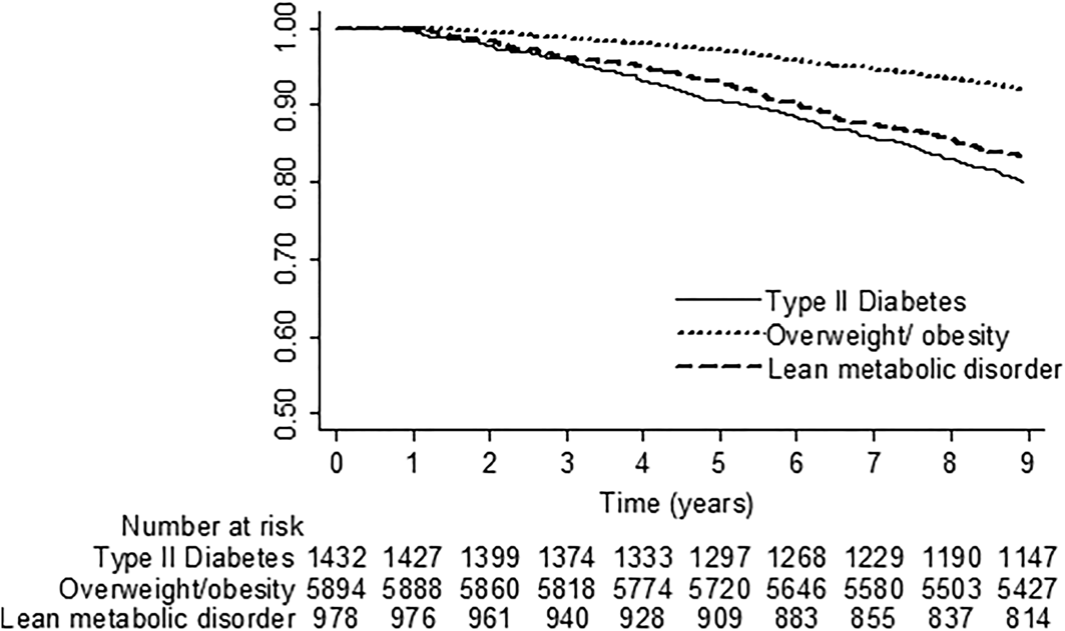
Overall survival of participants with MAFLD categorized by metabolic phenotypes.

The study cohort was followed up for an average of 8.52 ± 1.43 years (range 0.76–8.96). During the follow-up period of 71,145 person-years, 916 participants with MAFLD died, and the cumulative all-cause mortality rate was 12.88 per 1000 person-years. Individuals with MAFLD who exhibited the diabetes phenotype had a higher overall mortality rate compared to those who displayed the overweight/obesity phenotype (24.24 vs. 9.11 per 1000 person-years, HR 3.59, 95% CI 2.75–4.69; P < 0.001) (Fig. 2). Individuals with the lean metabolic phenotype had a significantly higher all-cause mortality rate compared to MAFLD subjects with the overweight/obesity phenotype (20.13 vs. 9.11 per 1000 person-years; HR 2.09, 95% CI 1.66–2.64; P < 0.001). Moreover, taking into account age, sex, education, current smoking, daily alcohol consumption, high LTPA, CCI, and handgrip strength, it was observed that subjects with the diabetes phenotype (aHR 1.59, 95% CI 1.18–2.13) and those with the lean metabolic phenotype (aHR 1.28, 95% CI 1.01–1.64) had a greater risk of all-cause mortality than those with the overweight/obesity phenotype.

### Alcohol consumption and the risk of all-cause mortality

**Figure 3:**
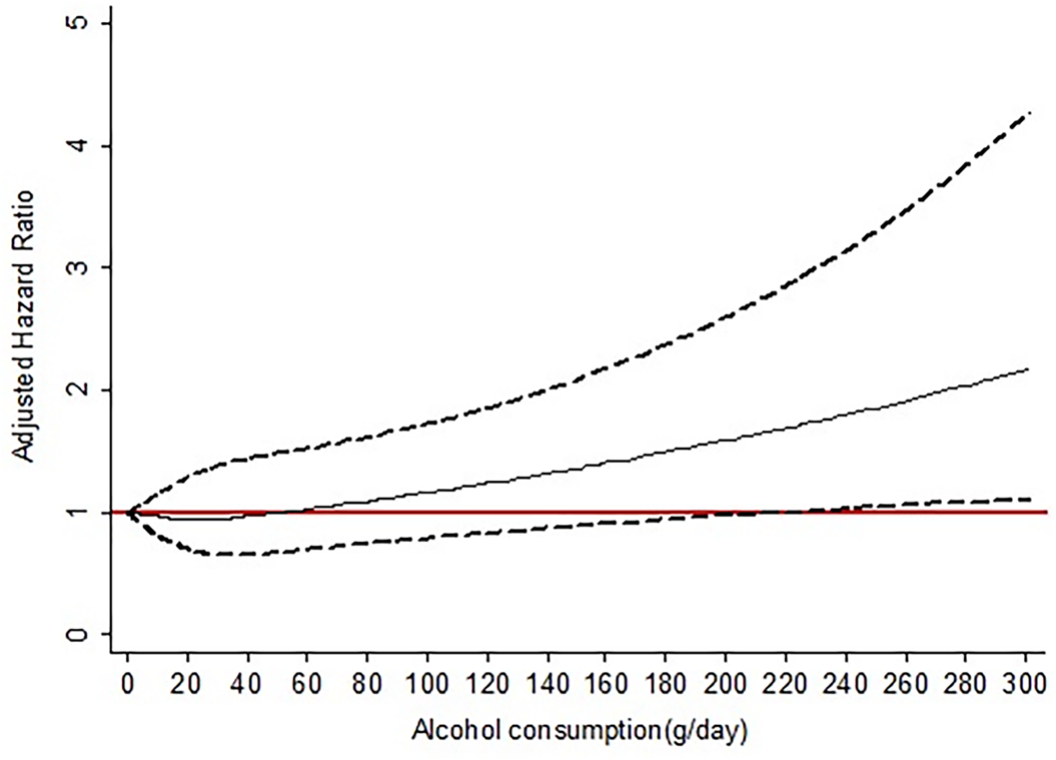
Spline adjusted hazard ratio graph of daily alcohol consumption on all-cause mortality in MAFLD.

The association between the continuous measure of alcohol consumption at baseline and fatal events is represented in Fig. 3. After adjusting for age, sex, education, smoking, high LTPA, CCI, and handgrip strength, there was a J-shaped association between daily alcohol consumption and the risk of overall mortality. The risk of death tended to increase when alcohol intake exceeds 50 g/day, compared to abstainers.

We further explored the baseline characteristics of the MAFLD population according to levels of alcohol consumption (Table 2). The proportions of participants who were abstainers or light drinkers, moderate drinkers, risk drinkers, and heavy drinkers were 72.2%, 11.0%, 8.8%, and 8.1%, respectively. We observed significant trends across strata of daily alcohol consumption for gender, education, smoking status, LTPA, and all metabolic risk factors. In particular, abstainers or light drinkers were more often female, had lower education attainment, were less often smokers, were less frequently engaged in high LTPA, and exhibited the highest prevalence of hyperglycemia and low HDL-C but the lowest prevalence of hypertriglyceridemia. Participants who were moderate drinkers had the lowest prevalence of hyperglycemia and hypertension. Heavy drinkers were more often male, had higher educational attainment, were often smokers, and exhibited the highest prevalence of hypertension, hypertriglyceridemia, and central obesity but had the lowest prevalence of low HDL-C.

**Table 2:** Baseline characteristics of MAFLD patients according to levels of daily alcohol consumption.

Characteristics	Levels of daily alcohol consumption				
	No-to-light consumption	Moderate consumption	Risk consumption	Heavy consumption	*P* for trend
Number (%)	5995 (72.2)	910 (11.0)	728 (8.8)	671 (8.1)	
Age (years)	49.8 ± 14.2	46.4 ± 10.4	44.9 ± 9.4	41.9 ± 9.2	0.086
Male gender, n (%)	1526 (23.7)	594 (62.9)	441 (57.7)	530 (81.5)	< 0.001
Education, n (%)					
Primary	474 (4.7)	38 (2.5)	22 (2.8)	13 (2.0)	< 0.001
Secondary	4243 (71.6)	579 (69.6)	443 (63.4)	336 (47.3)	< 0.001
Higher	1265 (23.7)	290 (27.9)	262 (33.9)	319 (50.7)	< 0.001
Body mass index (kg/m^2^)	27.1 ± 4.4	26.7 ± 3.2	26.4 ± 3.5	27.0 ± 3.4	0.645
Smoking status, n (%)					
Never	4786 (83.0)	450 (51.8)	370 (53.9)	244 (34.8)	< 0.001
Former	692 (7.6)	202 (16.8)	143 (17.7)	144 (21.7)	< 0.001
Current	517 (9.4)	258 (31.4)	215 (28.4)	283 (43.4)	< 0.001
High LTPA, n (%)	4,336 (79.8)	716 (84.9)	590 (85.5)	548 (85.3)	< 0.001
Charlson Comorbidity index	1.24 ± 0.61	1.13 ± 0.35	0.12 ± 0.34	0.12 ± 0.32	0.112
No. of metabolic risk factor	2.56 ± 1.07	2.32 ± 0.92	2.29 ± 0.89	2.39 ± 0.91	0.341
Metabolic risk factor, n (%)					
Hyperglycemia	2145 (28.4)	254 (20.3)	204 (24.2)	227 (27.1)	< 0.001
Hypertension	3740 (50.2)	499 (44.6)	404 (47.7)	398 (53.3)	< 0.001
Hypertriglyceridemia	3588 (60.2)	633 (71.6)	540 (76.4)	493 (76.9)	< 0.001
Low HDL-C	4090 (70.6)	508 (58.6)	384 (54.9)	331 (49.8)	< 0.001
Central obesity	4861 (81.2)	775 (82.4)	601 (80.6)	615 (88.7)	< 0.001
Glucose (mg/dL)	96.2 ± 34.1	94.2 ± 35.7	95.2 ± 35.1	94.9 ± 26.3	0.416
Total cholesterol (mg/dL)	219.9 ± 47.0	214.3 ± 40.9	217.9 ± 41.5	218.1 ± 37.2	0.860
LDL-C (mg/dL)	138.1 ± 42.9	128.0 ± 36.9	128.2 ± 39.1	127.1 ± 35.0	0.156
HDL-C (mg/dL)	43.4 ± 10.5	42.6 ± 8.6	44.0 ± 9.1	43.2 ± 8.8	0.805
Triglycerides (mg/dL)	195.1 ± 119.7	228.4 ± 119.3	241.9 ± 136.2	254.0 ± 136.4	0.094
Hemoglobin (g/dL)	13.0 ± 1.8	13.8 ± 1.4	13.6 ± 1.6	14.4 ± 1.5	0.102
Platelet (× 10^9^/cumm)	290.2 ± 78.1	280.0 ± 63.6	285.8 ± 63.0	278.9 ± 62.8	0.238
Creatinine (mg/dL)	0.86 ± 0.33	0.91 ± 0.21	0.88 ± 0.19	0.92 ± 0.16	0.223
Handgrip strength (kg)	27.9 ± 9.0	35.7 ± 8.7	34.4 ± 8.2	38.8 ± 8.2	0.124

BMI, body mass index; BP, blood pressure; LDL-C, low-density lipoprotein cholesterol; LTPA; leisure time physical activity; HDL-C, high-density lipoprotein cholesterol; MAFLD, metabolic-associated fatty liver disease.

Data are presented as the mean ± standard deviation or number (percentage).

Table 3 shows unadjusted incidence rates of death according to categories of alcohol consumption. The highest rate of fatal events occurred among subjects who were abstainers or light drinkers (14.46 per 1000 person-years of follow-up), and the lower rates were observed among subjects classified as risk drinkers or heavy drinkers (8.18 or 8.22 per 1000 person-years, respectively). After taking into account factors such as age, sex, education, smoking status, LTPA, CCI, and handgrip strength, it was found that moderate drinking was associated with a lower, but not statistically significant, risk of all-cause mortality (aHRs 0.63, 95% CI 0.32–1.23, P = 0.163) compared to abstinence or light drinking. On the other hand, risk drinking showed a tendency towards an increased risk of all-cause mortality (aHR 1.44, 95% CI 0.99–2.11, P = 0.057).

**Table 3:** The overall mortality of MAFLD participants stratified by levels of alcohol consumption.

Level of alcohol consumption	Deaths	Deaths per 1000 person-years	Age-and sex-adjusted HR (95% CI)	P-value	Multivariate model adjusted HR (95% CI)	P-value
No-to-light drinking	739	14.46	Reference		Reference	
Moderate drinking	77	9.82	0.56 (0.28–1.11)	0.094	0.63 (0.32–1.23)	0.163
Risk drinking	52	8.18	0.88 (0.57–1.34)	0.521	1.44 (0.99–2.11)	0.057
Heavy drinking	48	8.22	0.82 (0.41–1.66)	0.556	1.33 (0.70–2.52)	0.368

HR, hazards ratio; MAFLD, metabolic-associated fatty liver disease.

Age and sex adjusted HR (95% CI) was HR adjusted for age and sex.

Multivariate model was adjusted for age, sex, education, smoking status, leisure time physical activity, Charlson comorbidity index, and handgrip strength.

### Interaction between MAFLD phenotype and alcohol consumption on all-cause mortality risk

To assess whether alcohol consumption significantly influenced the association between MAFLD phenotype and overall mortality, interaction tests in multivariable models did not reveal evidence of interactions between MAFLD phenotype and alcohol drinking levels on mortality (P-value for interaction = 0.695).

**Figure 4:**
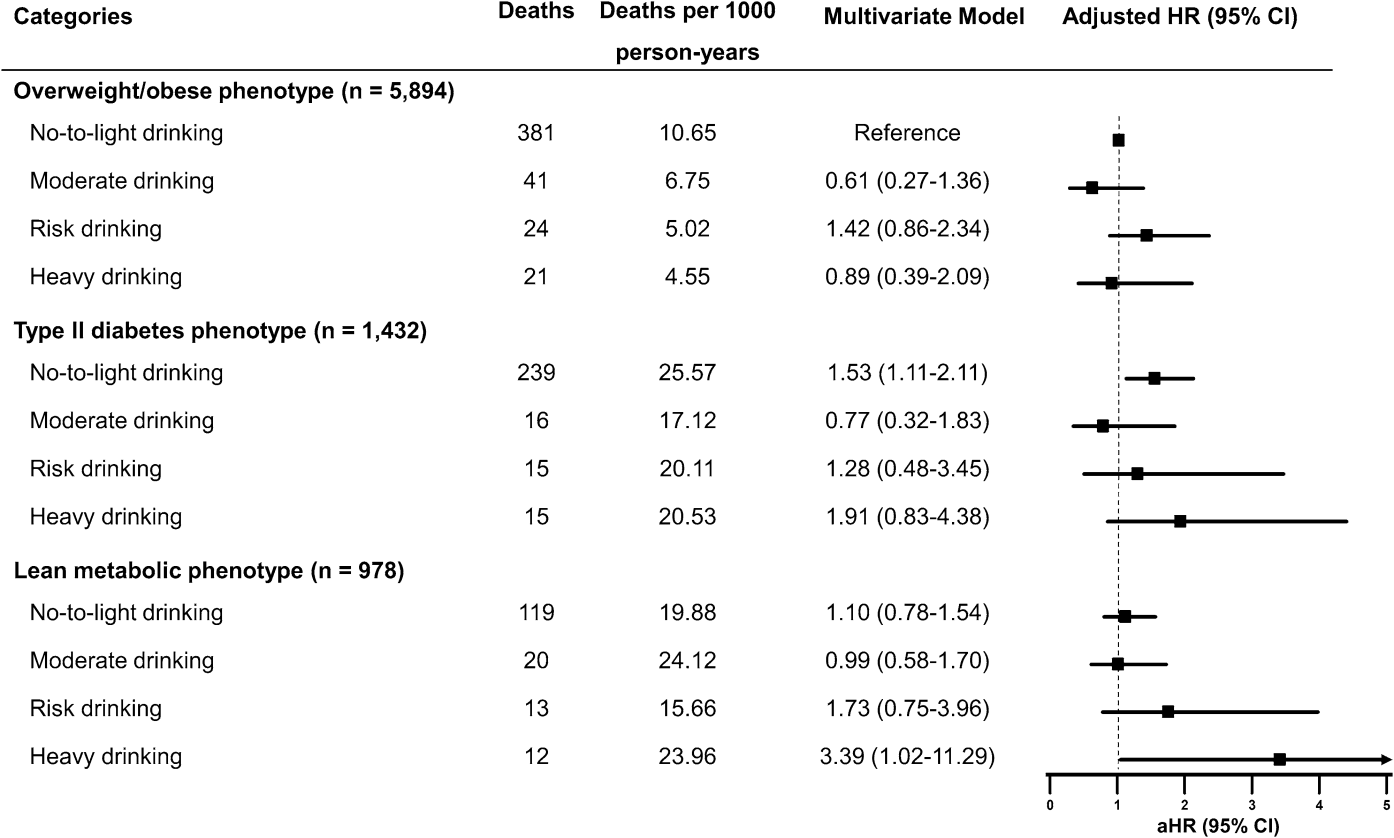
Interaction between metabolic phenotype and alcohol consumption on mortality Risk. Multivariate model was adjusted for age, sex, education, smoking status, leisure time physical activity, Charlson comorbidity index, and handgrip strength.

We further investigated the combined associations of metabolic phenotype and alcohol intake with mortality in subjects with MAFLD, conducting a stratified analysis of mortality risk for each metabolic phenotype based on daily alcohol intake (Fig. 4). Overweight/obese MAFLD subjects with abstinence or light drinking were used as the reference group. Among subjects with overweight/obese MAFLD, the risk of overall mortality for higher drinking categories did not significantly differ from abstinence or light drinking. For participants who abstained or consumed alcohol < 10 g/day, the mortality risk of individuals with the diabetes phenotype was significantly higher than the overweight/obese counterparts after multivariable adjustment (Fig. 4). Interestingly, there was an increased risk for all-cause mortality among individuals with the lean metabolic phenotype, reporting heavy alcohol consumption of > 50 g/day for women and > 60 g/day for men (aHR 3.39, 95% CI 1.02–11.29, P = 0.047).

### The risk of all-cause mortality between individuals with MASLD, MetALD, and ALD

Among 8635 participants with hepatic steatosis, 7127 individuals (83.0%) were classified as MASLD, 752 subjects (8.8%) as MetALD, and 708 persons (8.3%) as ALD. The remaining 48 subjects exhibited no cardiometabolic risk factors and no excessive alcohol intake. The baseline characteristics of subjects with MASLD, MetALD, and ALD are summarized in Table 4. Among the three groups, participants with MASLD were the oldest, predominantly female, had the highest CCI score, the lowest proportion of former and current smokers, the lowest daily alcohol intake, the highest prevalence of low HDL-C, and the lowest mean values of triglyceride, hemoglobin, and handgrip strength. Compared with the MASLD group, the MetALD group comprised younger individuals, with a higher proportion of males, lower mean BMI, higher education levels, higher LTPA, higher daily alcohol consumption, a higher proportion of former or current smokers, and hypertriglyceridemia, lower LDL-C levels, and higher values of hemoglobin and handgrip strength. Individuals with ALD were the youngest, predominantly male, exhibited the highest prevalence of current smokers, the highest daily alcohol intake, higher education levels, higher LTPA, the lowest CCI score, the lowest proportion of low LDL-C, and the highest mean levels of triglyceride, hemoglobin, and handgrip strength.

**Table 4:** Baseline characteristics of participants with MASLD, MetALD, and ALD.

Characteristics	Steatotic liver disease			P value	Multiple comparison
	MASLD^(a)^	MetALD^(b)^	ALD^(c)^		
Number (%)	7127 (83.0)	752 (8.76)	708 (8.3)		
Age (years)	49.3 ± 13.7	44.9 ± 9.4	41.6 ± 9.2	< 0.001	a ≠ b ≠ c
Male gender, n (%)	2185 (30.1)	450 (57.4)	555 (81.0)	< 0.001	a ≠ b ≠ c
Education, n (%)					
Primary	541 (4.6)	24 (2.8)	14 (1.9)	< 0.001	a ≠ b ≠ c
Secondary	4968 (71.0)	457 (63.3)	355 (47.8)		
Higher	1602 (24.3)	270 (34.0)	336 (50.4)		
Body mass index (kg/m^2^)	26.9 ± 4.2	26.3 ± 3.5	26.6 ± 3.5	0.012	a ≠ b, b ≠ c
Alcohol use (g/day)	3.4 ± 7.3	38.2 ± 8.9	132.8 ± 107.0	< 0.001	a ≠ b ≠ c
Smoking status, n (%)					
Never	5395 (77.8)	386 (53.8)	259 (34.8)	< 0.001	a ≠ b ≠ c
Former	922 (9.2)	144 (17.3)	150 (21.3)		
Current	810 (13.0)	222 (28.9)	299 (44.0)		
High LTPA, n (%)	5216 (80.7)	612 (85.7)	581 (85.2)	0.004	a ≠ b, a ≠ c
Charlson Comorbidity index	1.22 ± 0.57	0.12 ± 0.34	0.11 ± 0.31	< 0.001	a ≠ b, a ≠ c
Metabolic risk factor, n (%)					
Hyperglycemia	2404 (26.2)	204 (23.4)	228 (25.2)	0.586	
Hypertension	4294 (48.3)	406 (46.4)	404 (49.7)	0.476	
Hypertriglyceridemia	4315 (61.6)	554 (76.1)	518 (77.1)	< 0.001	a ≠ b, a ≠ c
Low HDL-C	4667 (67.2)	390 (54.2)	334 (46.2)	< 0.001	a ≠ b ≠ c
Central obesity	5713 (79.8)	609 (79.4)	630 (84.8)	0.196	
Glucose (mg/dL)	95.5 ± 34.2	91.7 ± 34.9	93.9 ± 25.3	0.194	
Total cholesterol (mg/dL)	219.1 ± 46.1	217.3 ± 41.4	217.0 ± 36.4	0.078	
LDL-C (mg/dL)	136.4 ± 42.1	127.6 ± 39.0	125.0 ± 34.8	< 0.001	a ≠ b, a ≠ c
HDL-C (mg/dL)	43.5 ± 10.3	44.1 ± 9.1	43.7 ± 8.7	0.227	
Triglycerides (mg/dL)	199.7 ± 119.8	240.4 ± 135.3	256.1 ± 134.6	< 0.001	a ≠ b ≠ c
Hemoglobin (g/dL)	13.2 ± 1.8	13.8 ± 1.6	14.3 ± 1.5	< 0.001	a ≠ b ≠ c
Platelet (× 10^9^/cumm)	288.8 ± 77.9	286.0 ± 62.9	277.9 ± 62.0	0.183	
Creatinine (mg/dL)	0.87 ± 0.31	0.88 ± 0.19	0.92 ± 0.16	0.665	
Handgrip strength (kg)	29.1 ± 9.4	34.3 ± 8.3	38.5 ± 8.0	< 0.001	a ≠ b ≠ c

ALD, alcohol-associated liver disease; BMI, body mass index; BP, blood pressure; LDL-C, low-density lipoprotein cholesterol; LTPA; leisure time physical activity; HDL-C, high-density lipoprotein cholesterol; MASLD, metabolic dysfunction-associated steatotic liver disease; MetALD, metabolic dysfunction- and alcohol-associated liver disease.

Data are presented as the mean ± standard deviation or number (percentage).

The χ^2^ test and one-way analysis of variance were used to compare the characteristics of the study participants. Post hoc multiple comparison analysis was performed with Bonferroni correction.

a ≠ b ≠ c: There is statistical difference among MASLD (a), MetALD (b), and ALD (c).

a ≠ b, a ≠ c: There is statistical difference between MASLD (a) and MetALD (b) and MASLD (a) and ALD (c), while there is no statistical difference between MetALD (b) and ALD (c).

b ≠ c: There is statistical difference between MetALD (b) and ALD (c).

a ≠ b, b ≠ c: There is statistical difference between MASLD (a) and MetALD (b) and between MetALD (b) and ALD (c), while there is no statistical difference between MASLD (a) and ALD (c).

Participants with MASLD, MetALD, and ALD exhibited overall mortality rates of 13.86, 7.91, and 7.94 per 1000 person-years, respectively. After adjusting for factors such as age, sex, education, smoking status, LTPA, CCI, and handgrip strength, it was observed that MetALD was significantly associated with an increased risk of all-cause mortality (aHRs 1.57, 95% CI 1.04–2.35, P = 0.032) compared with MASLD. ALD displayed a higher, albeit not statistically significant, risk of all-cause mortality (aHR 1.49, 95% CI 0.83–2.71, P = 0.174) compared with MASLD.

## Discussion

### Principle findings

In this longitudinal, nationally representative, population-based study, we found that MAFLD individuals with the diabetes or lean metabolic phenotypes had a higher risk of all-cause mortality compared to those who were overweight/obese. Notably, we observed a non-linear relationship between daily alcohol consumption and the risk of all-cause mortality in MAFLD patients. Heavy alcohol consumption, defined as daily consumption of more than 50 g/day for women and 60 g/day for men, increased the risk of all-cause mortality among MAFLD subjects with the lean metabolic phenotype. Additionally, individuals with MetALD, characterized by both risky alcohol consumption and metabolic dysfunction, exhibited a significantly higher risk of all-cause mortality compared to those with MASLD, while individuals with ALD showed a slightly elevated, albeit not statistically significant, risk.

### Interpretation

MAFLD was initially introduced as a fatty liver disease with concomitant manifestations of metabolic dysregulation^1^. It is now recognized as a heterogeneous disease with multiple metabolic phenotypes. Few studies have investigated mortality outcomes of MAFLD based on its diagnostic criteria. Hence, we categorized MAFLD participants into three metabolic phenotypes and assessed whether long-term mortality risks differed. Our analysis, focusing on the impact of metabolic phenotype at the MAFLD population level, uses patients with the overweight/obese phenotype as a reference group to assess the prognostic value of metabolic phenotypes. Intriguingly, our results indicate that the prognosis of MAFLD patients varies based on their metabolic phenotypes. Specifically, diabetes (aHR 1.59, 95% CI 1.18–2.13) and lean metabolic phenotypes (aHR 1.28, 95% CI 1.01–1.64) were independent predictors for all-cause mortality among patients with MAFLD. These findings support the idea that MAFLD patients have heterogeneous risks of overall mortality that vary according to accompanying metabolic dysregulation. Thus, classifying MAFLD subtypes based on the metabolic phenotype can potentially be used for risk stratification to promote early clinical interventions.

There is growing evidence showing that MAFLD and type 2 diabetes mellitus frequently coexist, potentiating adverse outcomes and individual progression of both disorders^16^. MAFLD patients with diabetes often exhibit higher severity and progression of MAFLD, leading to an increased risk of liver-related mortality^17–19^. Some studies with biopsy-proven MAFLD have identified diabetes as an independent risk factor for significant fibrosis^20^. Another histologic study revealed that non-alcoholic fatty liver disease, recently renamed MASLD, and type 2 diabetes had an additive effect on the development of aggressive outcomes such as cirrhosis and death^21^. Our study found that type 2 diabetes is an independent risk factor for mortality in MAFLD patients, increasing all-cause mortality by nearly 60% compared to non-diabetic patients with overweight or obesity. The connection between diabetes and mortality in MAFLD patients is intricate and seems to be related to insulin resistance. Our analysis revealed that MAFLD individuals with the diabetes phenotype were older and had more underlying comorbidities and metabolic risk factors such as hyperglycemia, hypertension, and low HDL-C, as well as higher serum creatinine levels compared with their overweight/obese counterparts. Considering these cardiometabolic risk factors in MAFLD individuals with diabetes, devastating microvascular and macrovascular complications of diabetes, such as cardiovascular disease and nephropathy, could be attributed to the increased risk of all-cause mortality in MAFLD patients with the diabetes phenotype.

The lean metabolic phenotype is one of the three defining criteria for MAFLD, and it plays a critical role in adverse clinical outcomes. Our study showed that 11.8% of the MAFLD cohort exhibited the lean metabolic phenotype using a conservative lower BMI for Asian populations. Our lean participants can be characterized as metabolically obese individuals with a normal weight, given their notably high prevalence of hypertriglyceridemia and low HDL-C. We found that MAFLD subjects with the lean metabolic phenotype were associated with a nearly 30% increased risk of all-cause mortality compared with those with overweight/obese MAFLD, independent of physiological, lifestyle, and comorbid factors. The prognosis of lean individuals with fatty liver disease remains subject to debate. Although NAFLD individuals with a lean body habitus have less severe liver disease than their obese counterparts, lean individuals still experience both hepatic and extrahepatic complications^22^. An updated meta-analysis of 14 observational studies revealed that lean MASLD was associated with a 1.6-fold increased long-term risk of all-cause mortality, independent of age, sex, and cardiometabolic risk factors^23^. The mechanisms underlying this heightened mortality risk among lean MAFLD patients remain unclear, but evidence suggests a higher risk of liver-related events and mortality^24,25^, potentially linked to genetic factors like the patatin-like phospholipase domain-containing 3 gene variant, particularly the GG genotype. Moreover, hypertriglyceridemia has been associated with MASLD progression in lean individuals, highlighting the importance of metabolic dysregulations in disease prognosis^26^. The relationship between hypertriglyceridemia and MASLD progression requires further investigation. Additionally, sarcopenia has emerged as a prognostic factor for all-cause mortality in MASLD patients, particularly in lean persons with metabolic dysregulations^27^. Handgrip strength, indicative of sarcopenia, appears to be more pronounced in lean individuals with metabolic dysregulations and may further contribute to the mortality risk in MAFLD patients with the lean metabolic phenotype. These findings underscore the complex interplay between metabolic factors, genetic predisposition, and sarcopenia in shaping the prognosis of lean individuals with MAFLD.

While regular alcohol drinking is common in the general population, its impact on overall health appears to intensify notably in individuals with metabolic risk factors^28^. However, existing epidemiological evidence regarding the relationship between alcohol intake and mortality risk among patients with MAFLD is limited. Some studies suggest that alcohol consumption may influence the prognosis of MAFLD^29,30^. Our comprehensive investigation aimed to elucidate the potential adverse effects of alcohol consumption in MAFLD patients, revealing a J-shaped association between alcohol consumption and overall mortality. Using Cox models with penalized splines, we observed that harmful alcohol consumption manifested after surpassing 50 g/day, while moderate drinking exhibited a favorable impact on metabolic traits such as hyperglycemia and hypertension, resulting in a reduced risk of overall mortality. These findings may be explained by the complex effects of alcohol on cardiovascular and liver health. Moderate alcohol drinking may confer cardiovascular benefits, but excessive alcohol intake can contribute to cardiovascular diseases and related mortality^31,32^, especially in patients with metabolic dysregulations like those seen in MAFLD. Additionally, a population-based cohort study in Finland demonstrates a J‐shaped association between alcohol consumption and all‐cause mortality, with a dose-dependent risk increase with alcohol intake for incident advanced liver disease and cancers^33^. However, when stratifying mortality risk according to alcohol intake categories, we found that subjects in higher drinking categories did not significantly differ in mortality risk from abstainers or light drinkers. This outcome may be due to the limited number of Thai participants consuming excessive alcohol, hindering our ability to estimate potential risks in these categories. Conflicting results from the US National Health and Nutrition Examination Survey III cohort regarding the detrimental effects of excessive alcohol consumption on MAFLD mortality risk may stem from differences in study design, particularly the use of participants without MAFLD as the reference and the oversight in categorizing daily alcohol consumption, thereby neglecting its nonlinear association with mortality risk^7^.

Several epidemiological studies have demonstrated that the combined effects of harmful drinking and metabolic dysfunction on liver disease are supra-additive^34,35^. A population-based study conducted in Finland revealed that weekly binge drinking (≥ 60 g per occasion) combined with metabolic syndrome had significant supra-additive effects on liver-related outcomes^36^. A study conducted in the US on adults with fatty liver disease showed that there is a combined effect of excessive drinking and metabolic syndrome on all-cause mortality^10^. Consistent with both studies, our investigation found that daily heavy drinking (> 50 g for women and > 60 g for men) was associated with a significantly higher risk of all-cause mortality among lean individuals with metabolic dysregulation. The risk is up to 3.39 times higher compared to abstainers or light drinkers with overweight/obesity. This finding suggests that heavy drinking and metabolic risk factors have a synergistic effect on mortality in lean patients with MAFLD. However, our analysis did not establish a synergistic relationship between excessive alcohol consumption and type 2 diabetes in increasing the risk of all-cause mortality. The physiological mechanisms that cause this phenomenon are intricate and not yet entirely comprehended, especially concerning how it affects only lean individuals. It is possible that the combination of metabolic dysfunction, excessive alcohol consumption, and other unhealthy lifestyle habits common in lean individuals with high-risk alcohol intake may result in worse clinical outcomes.

The Delphi consensus panel recently introduced a new nomenclature for fatty liver disease, aiming to eliminate stigmatizing terms and defining excessive liver fat accumulation as "steatotic liver disease (SLD)", subclassified into MASLD, MetALD, and ALD^13^. However, questions remain regarding the alcohol intake thresholds defined in this nomenclature. Our study explored the intricate relationship between alcohol consumption, metabolic risk factors, and all-cause mortality among individuals with different classifications of SLD. We observed distinct lifestyle and metabolic profiles among those classified into the MASLD, MetALD, and ALD groups. Notably, participants with MetALD exhibited a higher daily alcohol consumption compared to those with MASLD, while individuals with ALD displayed the highest daily alcohol intake. Our findings revealed a concerning trend in mortality rates among individuals with different classifications of SLD. Specifically, after adjusting for physiological, lifestyle, and comorbid factors, we found that individuals with MetALD had a significantly increased risk of all-cause mortality compared to those with MASLD. Although ALD subjects had a higher mortality risk, it did not reach statistical significance. This finding provides valuable insights into the complex interplay between alcohol intake and metabolic risk factors, which aligns with prior evidence of interactions between these factors on the risk of liver disease progression in patients with SLD^8,28,35^. Future research should focus on understanding mechanisms driving mortality outcomes, especially cardiovascular disease, cancers, and cirrhosis, to inform targeted interventions for this population.

The strengths of this study included the large nationally representative sample and comprehensive analysis of the effects of metabolic phenotypes and alcohol intake on mortality while controlling for demographic factors, education level (as a rough indicator of socioeconomic status), lifestyle behaviors, comorbidities, and a parameter of sarcopenia. Reporting of alcohol consumption at screening intended that there was no recall bias. However, certain limitations should be acknowledged. First, the diagnosis of fatty liver disease was based on the LAP score, which is suitable for large population-based studies when acquiring liver biopsy or imaging is not feasible. The LAP score has been validated across various ethnicities, including populations in the US, Europe, and Asia^37–42^. Second, this is an observational study, and daily alcohol consumption was self-reported via questionnaires administered by interviewers, potentially leading to underreporting of consumption levels. Nonetheless, this is unlikely, as we observed the association between higher categories of alcohol intake and lower proportions of low HDL-C, a biological marker of alcohol use^43^, thus supporting the reliability of self-reported alcohol intake. Third, all covariates were available only at baseline, so we were unable to capture changes in potential confounding factors over time, such as glycemic control, weight change, and drinking habits during follow-up. Finally, due to the restricted nature of the data, we could not evaluate cause-specific mortality as there was no follow-up data on disease-specific adverse events.

## Conclusion

This nationwide population-based study compared the characteristics and all-cause mortality of participants with MAFLD who were classified as metabolic phenotypes of diabetes, overweight/obese, and lean metabolic disorder. We discovered clinical and outcome differences between metabolic phenotypes, suggesting a heterogeneous disease of MAFLD. This finding highlights the importance of managing metabolic disorders while controlling liver disease progression. Our study has found that there is an interactive effect between alcohol consumption and metabolic risk factors in individuals with MAFLD, particularly in lean individuals and those with MetALD that increases the risk of all-cause mortality. Individuals who have both high alcohol consumption and metabolic risk factors are at a particularly high risk of death. Hence, further research is needed to identify effective interventions for reducing harm caused by metabolic dysfunctions and alcohol consumption in patients with MAFLD or SLD.

## Data Availability

The datasets generated and/or analysed during the current study are not publicly available due to the policy of the institutes, but are available from the corresponding author on reasonable request.
